# The impact of the COVID-19 pandemic on healthcare-associated infections in intensive care unit patients: a retrospective cohort study

**DOI:** 10.1186/s13756-021-00959-y

**Published:** 2021-06-04

**Authors:** V. Baccolini, G. Migliara, C. Isonne, B. Dorelli, L. C. Barone, D. Giannini, D. Marotta, M. Marte, E. Mazzalai, F. Alessandri, F. Pugliese, G. Ceccarelli, C. De Vito, C. Marzuillo, M. De Giusti, P. Villari

**Affiliations:** 1 Department of Public Health and Infectious Diseases, Sapienza University of Rome, Piazzale Aldo Moro 5, 00185 Rome, Italy; 2 Department of Anaesthesia and Intensive Care Medicine, Umberto I Teaching Hospital, Sapienza University of Rome, Rome, Italy; 3 Department of General and Specialist Surgery “P. Stefanini”, Sapienza University of Rome, Rome, Italy

**Keywords:** Healthcare-associated infection, Intensive care unit, COVID-19, SARS-CoV-2, Devices-related infections

## Abstract

**Background:**

During the intensive care units’ (ICUs) reorganization that was forced by the COVID-19 emergency, attention to traditional infection control measures may have been reduced. Nevertheless, evidence on the effect of the COVID-19 pandemic on healthcare-associated infections (HAIs) is still limited and mixed. In this study, we estimated the pandemic impact on HAI incidence and investigated the HAI type occurring in COVID-19 patients.

**Methods:**

Patients admitted to the main ICU of the Umberto I teaching hospital of Rome from March 1st and April 4th 2020 were compared with patients hospitalized in 2019. We assessed the association of risk factors and time-to-first event through multivariable Fine and Grey’s regression models, that consider the competitive risk of death on the development of HAI (Model 1) or device related-HAI (dr-HAI, Model 2) and provide estimates of the sub-distribution hazard ratio (SHR) and its associated confidence interval (CI). A subgroup analysis was performed on the 2020 cohort.

**Results:**

Data from 104 patients were retrieved. Overall, 59 HAIs were recorded, 32 of which occurred in the COVID-19 group. Patients admitted in 2020 were found to be positively associated with both HAI and dr-HAI onset (SHR: 2.66, 95% CI 1.31–5.38, and SHR: 10.0, 95% CI 1.84–54.41, respectively). Despite being not confirmed at the multivariable analysis, a greater proportion of dr-HAIs seemed to occur in COVID-19 patients, especially ventilator-associated pneumonia, and catheter-related urinary tract infections.

**Conclusions:**

We observed an increase in the incidence of patients with HAIs, especially dr-HAIs, mainly sustained by COVID-19 patients. A greater susceptibility of these patients to device-related infections was hypothesized, but further studies are needed.

**Supplementary Information:**

The online version contains supplementary material available at 10.1186/s13756-021-00959-y.

## Introduction

The coronavirus disease 2019 (COVID-19) pandemic has enormously impacted the healthcare systems globally [1]. Widespread and sustained transmission of SARS-CoV-2 has resulted in high rates of hospitalization, forcing rapid increases in total hospital capacities [2]. Healthcare facilities have quickly adapted to manage a sudden and unexpected influx of patients [3]. Due to the frequent requirement of ventilation support in COVID-19 patients [4], intensive care units (ICUs) are the hospital wards that may have suffered the most from the burden of the pandemic [5]. To deliver adequate care and handle the pressure during the emergency, most ICUs were reorganized [5]. Within this context, the considerable increase in ICU beds and supplies demand has led to a necessary reallocation of resources [6] that, coupled with a scarcity of healthcare personnel [7], may have negatively affected some traditional activities [8].

Among these, collateral damage to long-established infection control measures, such as the prevention of healthcare-associated infections (HAIs), may have occurred [9]. Focusing resources to primarily mitigate SARS-CoV-2 spread may have inadvertently reduced the attention to traditional HAI prevention programs in terms of lack of surveillance efforts, process measures and containment strategies [10]. Also, the COVID-19 response itself may have caused significant supply shortages of personal protective equipment [11], crucial for a successful HAI control [12, 13]. These factors, combined with the rapid upscaling of ICU capacity, reduced staff to patient ratios, greater length of stay, and higher complexity of patients may have contributed to an increased risk of infection from cross-contamination of microorganisms between patients [14, 15]. Not to mention the high selective antibiotic pressure that during the pandemic may have facilitated the insurgence of bacterial resistance [14].

Nevertheless, evidence on the impact of COVID-19 on HAIs is still limited [16] and mixed. On the one hand, some data suggest an incremental effect of the COVID-19 pandemic on HAI onset [17–19], but its real burden has yet to be quantified [14]. On the other hand, recent studies have pointed out a positive indirect and unintended role of the infection prevention and control strategies, adopted originally to contain the SARS-CoV-2 transmission, on HAI prevention [20, 21]. Additionally, despite the frequent use of multiple invasive devices in COVID-19 patients [4], most studies have focused on the evaluation of HAI’s causative microorganisms [9, 20, 22] and, to a lesser extent, on HAI type. The aim of this study is twofold: (i) to estimate the impact of the COVID-19 pandemic on the HAI incidence during the first phase of the emergency, contributing to an understanding of its indirect consequences on patients and healthcare systems; and (ii) to investigate the HAI type occurring in patients with the SARS-CoV-2 infection, providing considerations to guide clinicians in implementing targeted prevention strategies.

## Methods

### Patients and data collection

Data on ICU patients and HAIs were retrieved from the active HAI surveillance system that has been conducted since May 2016 in the main ICU of the Umberto I teaching hospital of Rome by the Department of Public Health and Infectious Diseases [23]. For this study, we retrospectively analysed patients admitted to the ICU from 1st March 2020 to 4th April 2020 (the day of ICU admission of the last COVID patient during the first phase of the emergency) and compared them to patients admitted to the ICU one year before (i.e., between 1st March 2019 and 4th April 2019). For both cohorts, the follow-up was extended up to the 15th of June 2019 and 2020, respectively. The detailed methodology of the surveillance system is described elsewhere [23]. Briefly, to provide standard diagnostic criteria for the identification of HAI, such a surveillance system is based on a protocol derived from the National Healthcare Safety Network protocol of the Center for Disease Control [24] and the European Center for Disease Prevention and Control [25]. All patients hospitalized in the ICU for at least two consecutive calendar days are monitored until their discharge from the ICU. Incidence of blood infections related with central lines (catheter-related bloodstream infections, CRBSIs), pneumonia associated with mechanical ventilation (ventilation-associated pneumonia, VAP), and urinary tract infections associated with bladder catheters (catheter-associated urinary tract infections, CAUTIs) that occur after 48 h from the device insertion is registered. The surveillance system also routinely stores data on the incidence of BSI of unknown origin (BUO) and surgical site infections (SSIs) that occur 48 h after ICU admission or within 30 days after surgery, respectively. As for BUO, they are defined as laboratory confirmed BSIs that are not secondary to an infection at another body site [25], whereas SSIs are infections that occur near or at the incision site and/or deeper underlying tissue spaces [24].

Data are collected systematically using a form with four sections: (1) patient demographics and information on hospitalization (date of ICU admission, type of ICU admission, discharge date, status of the patient at discharge, pre-existing comorbidities); (2) exposure to risk factors: start and end date of the patient’s exposure to urinary catheterization, central venous catheterization, and mechanical ventilation. It is also specified whether the device was present within the 48 h prior to the onset of infection; (3) antibiotic therapy: information on the drug(s) used, start and end date of antibiotic therapy for each drug used; (4) diagnosed HAIs and microbiological cultures performed: site of infection, date of HAI onset and microbiological confirmation (date of sample collection and microorganisms identified). As for COVID-19 patients, laboratory confirmation of SARS-CoV-2 was defined as a positive result of real-time reverse transcriptase-polymerase chain reaction assay of nasal and pharyngeal swabs. We coded antibiotic consumption as having used any antibiotic agent for at least two days in a systemic administration (i.e., enteral or parenteral) for a different clinical reason than the first HAI in the time period from ICU admission to the day before the HAI onset or to the date of discharge.

The institutional ethics board of the Umberto I teaching hospital of Rome approved this study.

### Statistical analysis

Descriptive statistics were obtained using median and interquartile range (IQR) for continuous variables and proportions for dichotomous and categorical variables. The ICU mortality rate and the associated 95% confidence interval (CI) was calculated on 1000 patient-days. Two different outcomes were considered: HAI and device-related HAI (i.e., only VAP, CRBSI and CAUTI). Time-to-first event was estimated through survival analysis. Given the competitive risk of death, the Kaplan–Meier estimate of the survival function would have resulted in upward biases of the incidence function estimation [26]. Hence, we used competing risk modelling to explore the effect of the exposure of interest on the outcome incidence. Firstly, we estimated the cumulative incidence function (CIF) for each year of hospitalization (2019 vs. 2020). Secondly, the association between this variable and time-to-first event was assessed through multivariable Fine and Grey’s regression models for proportional hazard, that provided estimates of the sub-distribution hazard ratio (SHR) (i.e., the relative change in the instantaneous rate of the occurrence of the event in those subjects who are event-free or who have experienced the competitive event) [26, 27] and its associated 95% CI. Specifically, two Fine and Grey’s models were built to regress the SHR of first HAI (Model 1) and first device related-HAI (dr-HAI, Model 2). The main exposure of interest was adjusted for the same covariates in both analyses by including the potential confounders of the association, such as age and gender, based on expert knowledge [28]. Among the pre-existing conditions, only the most prevalent comorbidities were considered (i.e., hypertension and diabetes mellitus). Since days of central venous catheterization, days of urinary catheterization, and days of mechanical ventilation were highly collinear (variance inflation factor > 2.5), only the latter was kept for further analyses. As a result, the final models included the following variables: year of hospitalization (2020 vs. 2019), age (years, continuous), gender (female/male), hypertension (yes/no), diabetes mellitus (yes/no), and mechanical ventilation (days, continuous). Interaction terms between the variables were tested and were not significant considering 0.05 as cut-off. The proportionality assumption was checked by testing the statistical significance of interaction terms involving failure time, each one at a time.

A subgroup analysis was performed in the 2020 cohort, distinguishing patients with and without COVID-19. We used the same methods and variable selection process of the main analyses. Specifically, the CIFs were used to estimate the incidence of both outcomes (first HAI and first dr-HAI) comparing the two groups. Then, two multivariable Fine and Grey’s regression models were built to assess the association between the main exposure (i.e., being a COVID-19 patient) and time-to-first event (Model 3 for HAI, and Model 4 for dr-HAI, respectively).

All analyses were performed using STATA (StataCorp LLC, 4905 Lakeway Drive, College Station, 322 Texas, USA), version 15.1. A two-sided *p *value < 0.05 was considered statistically significant.

## Results

### Description of the cohorts

Data from 104 patients were analyzed: 42 hospitalized in 2019 and 62 hospitalized in 2020 (Table 1).

**Table 1:** Characteristics of the patients admitted to the main Intensive Care Unit (ICU) of Umberto I teaching hospital of Rome between 1st March and 4th April 2019 and 1st March and 4th April 2020. Results are expressed as number (percentage), median (interquartile range) or mean (standard deviation)

	2019 cohort	2020 cohort
Patients	42	62
Observation time, person-days	1130	1000
Gender (male)	24 (57.1)	41 (66.1)
Age, years	64.5 (52–76)	70 (61–79)
Admission to the ICU		
Other ward	11 (26.2)	31 (50.0)
Other hospital	2 (4.8)	5 (8.1)
Emergency Department	29 (69.0)	26 (41.9)
Coexisting conditions		
Hypertension	22 (52.4)	27 (43.6)
Diabetes mellitus	10 (23.8)	10 (16.1)
Asthma	1 (2.4)	4 (6.5)
Coronary heart disease	2 (4.8)	11 (17.7)
Chronic kidney disease	3 (7.1)	5 (8.1)
Chronic liver disease	2 (4.6)	0 (0.0)
Active cancer	4 (9.5)	7 (11.3)
Immunodeficiency	5 (11.9)	1 (1.6)
ICU deaths	10 (23.8)	35 (56.5)
Mortality rate (95% CI) per 1000 patient-days	8.8 (4.8–15.0)	35.0 (25.8–46.4)
Length of ICU stay, days	14.5 (7–36)	13 (6–21)
Central venous catheter, days	15.5 (7–33.5)	14 (6–23)
Urinary catheter, days	14.5 (7–35)	13 (6.5–22)
Invasive ventilation, days	13 (4–35)	8 (5–19)
Patients with invasive ventilation	38 (90.5)	53 (85.5)
Patients with at least one HAI	11 (26.2)	27 (43.6)
Patients with at least one dr-HAI	2 (4.8)	20 (32.3)
HAI per patient	0.33 (0.61)	0.73 (0.93)
dr-HAI per patient	0.05 (0.22)	0.44 (0.69)
HAI per infected patient	1.27 (0.48)	1.67 (0.62)
dr-HAI per infected patient	1.0 (0.0)	1.35 (0.49)
Antibiotic consumption before the first HAI		
Carbapenems	40 (95.2)	51 (82.3)
Extended spectrum cephalosporins	15 (35.7)	6 (9.7)
Glycopeptides	14 (33.3)	29 (46.8)
Macrolides	3 (7.1)	15 (24.2)
Penicillins + beta lactamase inhibitors	21 (50.0)	31 (50.0)

ICU: Intensive Care Unit. HAI: Healthcare-Associated Infection. dr-HAI: device-related Healthcare-Associated Infection. CI: Confidence Interval

Cumulative observation time from ICU admission to end of follow-up was 1130 patient-days and 1000 patient-days for the 2019 and 2020 cohort, respectively. Most patients were men, especially in the 2020 cohort (66.1%), that also had a higher median age (70 vs. 64.5 years). In 2019, patients admitted to the ICU came mostly from the Emergency Department (69.0%), while in 2020 patients were transferred from other wards more frequently (50.0%). Hypertension was the most prevalent comorbidity in both groups, followed by diabetes mellitus and coronary heart disease. In the 2019 cohort, at the end of a median length of stay of 14.5 days (IQR: 7–36), four patients were still in the ICU and ten had died (23.8%), accounting for an ICU mortality rate of 8.8 (95% CI 4.8–15.0) per 1000 patients-days. By contrast, in 2020, after a median follow-up time of 13 days (IQR: 6–21), one patient was still under observation and 35 had died, for a corresponding ICU mortality rate of 35.0 (95% CI 25.8–46.4) per 1000 patients-days. As for invasive devices, patients hospitalized in 2019 had a higher median use of central venous catheter, urinary catheter, and duration of mechanical ventilation. Also, more patients needed invasive respiratory support. In total, eleven patients (26.2%) experienced at least one HAI and two patients at least one dr-HAI (4.8%) in 2019, but these proportions were greater in 2020. Also, the average number of both HAI and dr-HAI per patient was higher in 2020, similarly to the average number of HAI and dr-HAI per infected patient. Antibiotic consumption before the first HAI was high in both cohorts, especially for carbapenems (95.2% and 82.3% of the patients, respectively) and penicillins (50.0% of the patients in both cohorts).

Overall, the active surveillance system registered 14 HAIs in 2019 and 45 infections in 2020 (Table 2). In 2019, most infections were BUO (85.8%), with only two device-related HAIs (i.e., one CRBSI and one CAUTI). By contrast, in 2020, the HAIs registered were mainly VAP (37.8%), followed by BUO (31.1%), CAUTIs (22.2%), *Clostridium difficile* infections (4.4%), healthcare-associated pneumonia (HAP, 2.2%) and SSI (2.2%). These infections were sustained by 81 microorganisms (19 in 2019 and 62 in 2020), among which *Acinetobacter baumannii* isolates were the majority in both years (31.6% in 2019, 29.0% in 2020), followed by *Enterococci* in 2019 (31.6%) and *Klebsiella pneumoniae* and other *Enterobacteriaceae* in 2020 (14.5% each).

**Table 2:** Type and frequency of all healthcare-associated infections (HAIs) registered by the active surveillance system among the patients admitted to the main Intensive Care Unit (ICU) of Umberto I teaching hospital of Rome between 1st March and 4th April 2019 and 1st March and 4th April 2020. Results are expressed as number (percentage)

	2019 cohort	2020 cohort
HAI	14 (100)	45 (100)
Device-related HAI		
VAP	0 (0.0)	17 (37.8)
CRBSI	1 (7.1)	0 (0.0)
CAUTI	1 (7.1)	10 (22.2)
BUO	12 (85.8)	14 (31.1)
* Clostridium difficile* infection	0 (0.0)	2 (4.4)
Surgical site infection	0 (0.0)	1 (2.2)
Healthcare-associated pneumonia	0 (0.0)	1 (2.2)
Microorganism	19 (100)	62 (100)
* Acinetobacter baumannii*	6 (31.6)	18 (29.0)
* Candida albicans* or *parapsilosis*	0 (0.0)	6 (9.7)
* Clostridium difficile*	0 (0.0)	2 (3.2)
* Enterobateriaceae*	3 (15.8)	9 (14.5)
* Enterococci*	6 (31.6)	3 (4.8)
* Klebsiella pneumoniae*	3 (15.8)	9 (14.5)
* Pseudomonas aeruginosa*	1 (5.3)	5 (8.1)
* Staphylococcus aureus*	0 (0.0)	2 (3.2)
Coagulase Negative *Staphylococci*	0 (0.0)	8 (12.9)

*VAP* Ventilator-Associated Pneumonia, *CRBSI* Catheter-Related Blood Stream Infection, *CAUTI* Catheter-Associated Urinary Tract Infection, *BUO* Bloodstream infections of Unknown Origin

### Main analyses

**Fig. 1:**
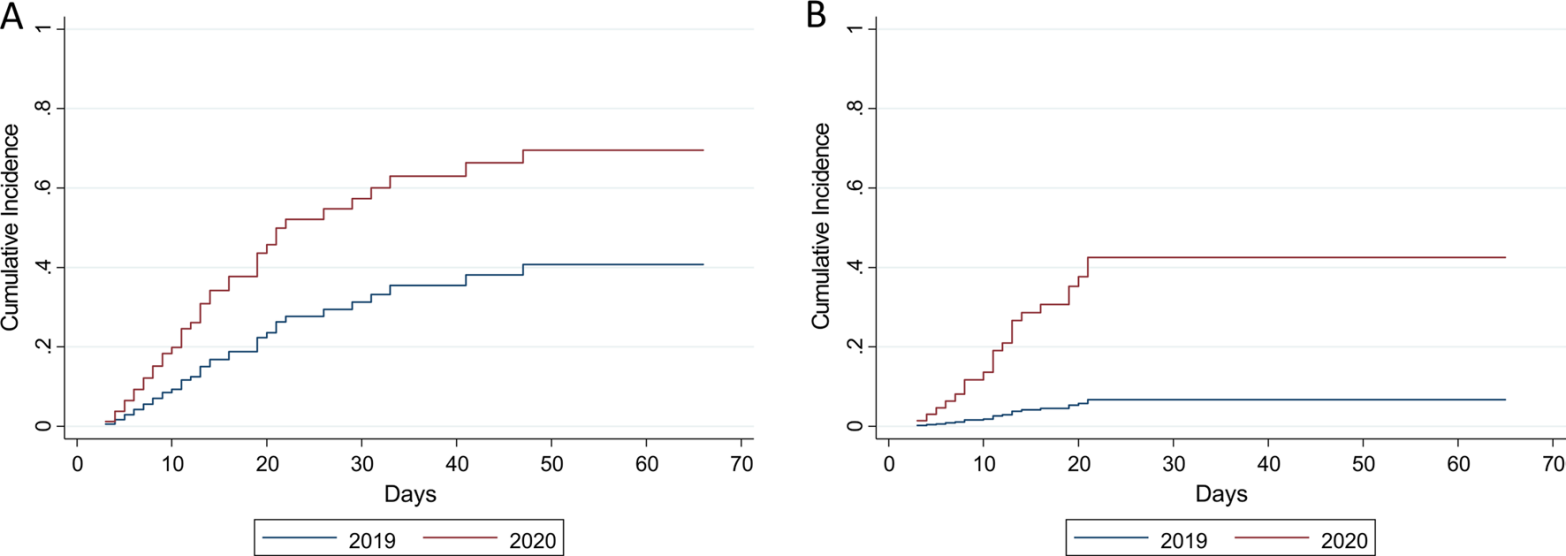
Cumulative incidence function for first healthcare-associated infection (**a**) or first device-related healthcare-associated infection (**b**) among the patients admitted to the main Intensive Care Unit (ICU) of Umberto I teaching hospital of Rome between 1st March and 4th April 2019 and 1st March and 4th April 2020

In 2020, an increased number of patients developed at least one HAI and dr-HAI (SHR: 2.27, 95% CI 1.19–4.34, and SHR: 7.96, 95% CI 1.34–34.38) (Fig. 1).

These associations were maintained at the multivariable analyses where patients admitted in 2020 were found to be positively associated with both HAI and dr-HAI (SHR: 2.66, 95% CI 1.31–5.38, and SHR: 10.0, 95% CI 1.84–54.41, respectively) (Table 3).

**Table 3:** Multivariable competing risk Fine-Gray regression models for first healthcare-associated infection (HAI, Model 1) or first device-related healthcare-associated infection (first dr-HAI, Model 2) among the patients admitted to the main Intensive Care Unit (ICU) of Umberto I teaching hospital of Rome between 1st March and 4th April 2019 and 1st March and 4th April 2020

	HAI (model 1)		dr-HAI (model 2)	
	SHR (95% CI)	*p* value	SHR (95% CI)	*p* value
Year of admission to the ICU (2020)	2.66 (1.31–5.38)	0.007	10.0 (1.84–54.41)	0.008
Sex (female)	0.93 (0.47–1.85)	0.835	0.77 (0.30–1.99)	0.585
Age (years)	0.97 (0.96–0.99)	0.006	0.97 (0.94–0.99)	0.016
Hypertension (yes)	2.83 (1.37–5.82)	0.005	1.63 (0.73–3.61)	0.184
Diabetes mellitus (yes)	1.88 (0.76–4.64)	0.169	1.51 (0.43–5.29)	0.522
Mechanical ventilation (days)	0.89 (0.84–0.94)	< 0.001	0.78 (0.65–0.93)	0.006
Mechanical ventilation*time	1.00 (1.002–1.006)	< 0.001	1.01 (1.003–1.025)	0.007

*SHR* sub-distribution hazard ratio, *CI* confidence interval, * interaction term

While being female did not seem to influence any outcome, being older was associated with a reduction in the sub-distribution hazards in both models (SHR: 0.97, 95% CI 0.96–0.99, and SHR: 0.97, 95% CI 0.94–0.99, respectively). As for the comorbidities, only hypertension was associated with HAI onset (SHR: 2.83, 95% CI 1.37–5.82). Exposure to mechanical ventilation seemed to have a time-varying association in both models (Table 3).

### Subgroup analyses

Out of the 62 patients hospitalized in 2020, 41 had COVID-19 (Additional file 1: Table S.1). Cumulative observation time from ICU admission to end of follow-up was 657 patient-days and 343 patient-days for patients with and without the SARS-CoV-2 infection, respectively. Overall, patients with COVID-19 were more likely to be male and older. As for the ICU admission, patients were mostly transferred from other wards in both groups. Hypertension was the most common comorbidity. At the time of censoring, 27 patients had died in the COVID-19 cohort and eight had died in the other group, accounting for an ICU mortality rate of 41 (95% CI 28–59) and 23 (95% CI 12–46) per 1000 patient-days, respectively. For COVID-19 patients, the median length of ICU stay was more than twice as long, as well as the median days of central venous catheter; higher was also the median days of urinary catheter use and invasive ventilation for intubated patients, with more patients undergoing mechanical ventilation. Over the ICU stay, patients in the COVID-19 cohort were more affected by HAIs than the other group: almost half of them developed at least one HAI and 39% at least one dr-HAI. Also, the COVID-19 cohort seemed to experience a higher number of infections per patient on average, a higher number of dr-HAI per infected patient on average but a lower number of HAI per infected patient. Regarding antibiotic use before the first HAI, for the two more utilized classes in 2020 (carbapenems and penicillins) consumption was substantially higher in the COVID-19 cohort.

In total, 32 infections (71.1%) were registered in SARS-CoV-2 positive patients (Additional file 1: Table S.2). Most infections were device-related, specifically VAP (46.9%) or CAUTIs (21.9%), followed by nine BUO (28.1%) and one HAP (3.1%). By contrast, patients without COVID-19 developed mainly BUO (38.5%), followed by CAUTIs (23.1%), VAP (15.4%), *Clostridium difficile* infections (15.4%) and SSIs (7.7%). These infections were sustained by 62 microorganisms (44 in patients with COVID-19 and 18 in patients without COVID-19), and *Acinetobacter baumannii* isolates were the majority in both (29.5% in the first group, and 27.7% in the other group), followed by *Enterobacteriaeae* in the COVID-19 cohort (18.2%) and *Klebsiella pneumoniae* and Coagulase Negative *Staphylococci* in the other cohort (16.7% each).

**Fig. 2:**
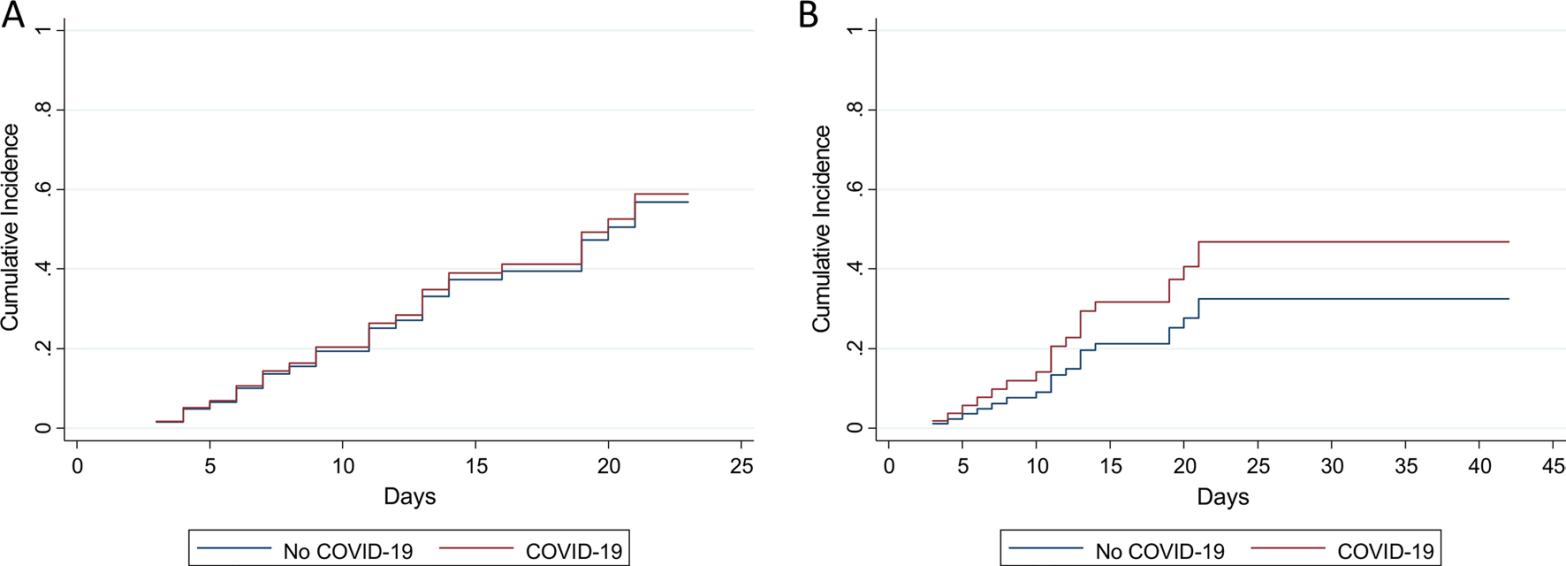
Cumulative incidence function for first healthcare-associated infection (**a**) or first device-related healthcare-associated infection (**b**) among the patients admitted to the main intensive care unit of Umberto I teaching hospital of Rome between 1st March and 4th April 2020

Figure 2 shows the CIFs for first HAI (SHR: 1.06, 95% CI 0.48–2.35) and first dr-HAI (SHR: 1.60, 95% CI 0.55–4.67) comparing patients with and without COVID-19.

At multivariable analyses, there was no difference in the sub-distribution hazards between the two groups for neither HAI nor dr-HAI, (SHR: 1.21, 95% CI 0.48–3.08 and SHR: 2.35, 95% CI 0.85–6.45) (Table 4).

**Table 4:** Multivariable competing risk Fine-Gray regression models for first healthcare-associated infection (HAI, Model 3) or first device-related healthcare-associated infection (dr-HAI, Model 4) among the patients admitted to the main intensive care unit of Umberto I teaching hospital of Rome between 1st March and 4th April 2020

	HAI (model 3)		dr-HAI (model 4)	
	SHR (95% CI)	*p *value	SHR (95% CI)	*p *value
COVID-19 (yes)	1.21 (0.48–3.08)	0.683	2.35 (0.85–6.45)	0.099
Sex (female)	0.86 (0.36–2.05)	0.730	0.80 (0.29–2.17)	0.660
Age (years)	0.98 (0.96–1.00)	0.068	0.96 (0.94–0.99)	0.007
Hypertension (yes)	2.04 (0.91–4.57)	0.082	1.65 (0.70–3.92)	0.255
Diabetes mellitus (yes)	1.41 (0.48–4.12)	0.529	1.16 (0.31–4.33)	0.826
Mechanical ventilation (days)	0.71 (0.57–0.88)	0.002	0.97 (0.91–1.03)	0.262
Mechanical ventilation*time	1.02 (1.01–1.04)	0.009	–	–

*SHR* sub-distribution hazard ratio, *CI* confidence interval, * interaction term

Older age was associated with a reduction in the hazards of dr-HAI only (SHR: 0.96, 95% CI 0.94–0.99). Sex did not seem to be an independent predictor of neither HAI nor dr-HAI, as well as hypertension and diabetes mellitus. Lastly, exposure to mechanical ventilation had a time-varying association with first HAI onset and no influence on first dr-HAI (Table 4).

## Discussion

In this study, we found an increase in the incidence of patients with HAIs and dr-HAIs during the first phase of the emergency, similarly to other findings [17, 18, 29]. These results may confirm the impact of the organizational challenges experienced during the COVID-19 pandemic that may have limited the traditional HAI’s prevention and control efforts [17]. Indeed, although several initiatives aimed at reducing SARS-CoV-2 spread may have increased awareness on infection prevention measures [21], many healthcare facilities had to contend with physical space limitations, constrained availability of personnel, shortages in personal protective equipment, and a large number of patients [30], as may have happened in our ICU, where the number of hospitalizations increased by almost 50%. Additionally, given the fear of getting infected [10], healthcare workers (HCWs) may have applied measures to primarily protect themselves, reducing the compliance to hygiene precautions and increasing the risk of cross-contamination [31] or minimizing the contact time with COVID-19 patients and thus facilitating the microorganisms’ growth [14]. However, further investigation is needed to quantify the specific impact that all the discussed factors may have had on HAI onset [14].

As for patients with and without COVID-19, it seemed that a higher proportion of HAIs, mainly dr-HAIs, occurred in the first group. Whereas at the multivariable analyses the incidence of patients with HAIs did not seem to differ across the two groups, the lack of difference in the incidence of patients with dr-HAIs was probably due to insufficient statistical power. Nonetheless, as consistently reported in the literature [32, 33], our COVID-19 patients required intense levels of support, with prolonged exposure to invasive devices coupled with high mortality rates, both indications of their severe clinical conditions [29, 34]. Conversely, SARS-CoV-2 negative patients shown shorter length of stay and use of invasive devices in comparison to the previous year, that might be explained by the change in the ward case mix led by the lower proportion of ICU admissions from the ED recorded in 2020. As happened in other countries [35, 36], the decreased opportunity for injury due to motor vehicle collision during the lockdown period may have reduced the number of ED visits for traumatic conditions with a consequent reduction in the care acuity of this group.

As for the HAI type, the highest impact of the pandemic was expected on central line-associated BSI (CLABSI) rates [14], defined as a primary BSI occurring in a patient that has a central line within the 48 h before the BSI development and is not bloodstream related to an infection at another site [24]. Hence, it is a more lenient diagnosis compared to the CRBSI that requires instead specific laboratory testing to identify the catheter as the source of the BSI [24]. Since our surveillance system collects data on CRBSI only, it could explain why we did not record any increase. Another hypothesis could be that our ICU staff is particularly experienced in placing and managing central lines, as demonstrated by the CRBSI low incidence in 2019, thanks to a few training interventions that were conducted in 2018 to reduce the CRBSI burden. Also, they do not use the femoral site, usually associated with a higher risk of CLABSI [37]. Likewise, we did not observe any substantial change in the number of BUO between the two years, that may be due to its stringent diagnosis (i.e., it is considered after exclusion of all potential infection sources), but further investigations should be conducted, since the topic is still under-investigated to date [38], and most studies do not specifically focus on BUO rates [15, 39]. By contrast, the VAP incidence increased from 0% in 2019 to almost 38% in 2020, becoming the most represented infection in the COVID-19 cohort. Current literature has already argued that, despite the challenges related to its diagnosis [40], these patients may be at higher risk [41, 42]. The reason for such a susceptibility could be the impaired immune cell function and the damage to the alveolar membrane, particularly strong in COVID-19 patients [43, 44]. Additionally, the micro-aspirations caused by prone positioning and the acute respiratory distress syndrome are both established VAP risk factors [42], similarly to older age and prolonged exposure to invasive ventilation, that in our study seemed to be protective probably as a result of the depletion of susceptible patients, that may have made the profile of the survivors apparently more favorable. Other pathophysiological mechanisms, such as the increased risk of traumas during prone positioning [14, 45], the prolonged exposure to urinary catheter, and the HCWs’ hesitancy to remove unnecessary devices to reduce the SARS-CoV-2 self-exposure [14], may have favored the CAUTI onset. Hence, as the pandemic continues, clinicians should enhance focus and implementation of evidence-based practices for dr-HAI prevention like bundles and checklists [14], especially in reorganized settings as the ICUs where new staff may lack specific training [46].

This study has several strengths and limitations. The main strength is our data comparability over time since information on patients and HAIs were collected as part of a 3-year surveillance system routinely carried out, meaning that the potential distortion of the results due to the ICU staff’s work overload is unlikely. Also, to the best of our knowledge, this is the first study that accurately distinguishes the HAI types and considers the competitive risk of death on HAI occurrence for patients with and without COVID-19. By contrast, the first limitation is represented by the small sample size that may have led to reduced statistical power, especially in the subgroup analyses. Secondly, the patients discharged from the ICU were no longer under surveillance, but only the most stable patients were chosen for transfer. Thirdly, even though we adjusted for pre-existing comorbidities, use of invasive device and COVID-19 infection, we may have not fully accounted for clinical severity, meaning that some residual confounding may be still present. Lastly, our analyses did not allow to confirm the greater susceptibility of COVID-19 patients to device-related infections. Further studies are needed to explore this issue.

## Supplementary Information

**Additional file 1: Table S.1**. Characteristics of the patients admitted to the main intensive care unit of Umberto I teaching hospital of Rome between 1st March and 4th April 2020. Results are expressed as number (percentage), mean (standard deviation) or median (interquartile range). **Table S.2**. Type and frequency of all healthcare-associated infections (HAIs) registered by the active surveillance system among the patients admitted to the main intensive care unit of Umberto I teaching hospital of Rome between 1st March and 4th April 2020. Results are expressed as number (percentage).

## Data Availability

The datasets used and/or analysed during the current study are available from the corresponding author on reasonable request.

## Abbreviations

ICU: Intensive care unit
HAI: Healthcare-associated infection
dr-HAI: Device-related healthcare-associated infections
CRBSI: Catheter-related blood stream infections
VAP: Ventilator-associated pneumonia
CAUTI: Catheter-associated urinary tract infection
BUO: Bloodstream infection of unknown origin
SSI: Surgical site infection
ED: Emergency Department
