# Supplementary material for: The impact of the COVID-19 pandemic on healthcare-associated infections in intensive care unit patients: a retrospective cohort study
Source: Antimicrob Resist Infect Control. 2021 Jun 4;10:87. doi: 10.1186/s13756-021-00959-y (PMC8177262; doi:10.1186/s13756-021-00959-y)
Supplement: Supplementary file 1 — Additional file 1: Table S.1. Characteristics of the patients admitted to the main intensive care unit of Umberto I teaching hospital of Rome between 1st March and 4th April 2020. Results are expressed as number (percentage), mean (standard deviation) or median (interquartile range). Table S.2. Type and frequency of all healthcare-associated infections (HAIs) registered by the active surveillance system among the patients admitted to the main intensive care unit of Umberto I teaching hospital of Rome between 1st March and 4th April 2020. Results are expressed as number (percentage). [file 13756_2021_959_MOESM1_ESM.docx]

Table S1. Characteristics of the patients admitted to the main intensive care unit of Umberto I teaching hospital of Rome between 1^st^ March and 4^th^ April 2020. Results are expressed as number (percentage), median (interquartile range) or mean (standard deviation).

|  | | 2020 cohort | |
| --- | --- | --- | --- |
|  |  | With COVID-19 | Without COVID-19 |
| Patients | | 41 | 21 |
| Observation time, person-days | | 657 | 343 |
| Gender (male) | | 30 (73.2) | 11 (52.4) |
| Age, years | | 72 (66-80) | 69 (52-76) |
| Admission to the ICU | |  |  |
|  | Ward | 17 (41.5) | 14 (66.7) |
|  | Other hospital | 5 (12.2) | 0 (0.0) |
|  | Emergency Department | 19 (46.3) | 7 (33.3) |
| Coexisting conditions | |  |  |
|  | Hypertension | 21 (51.2) | 6 (28.6) |
|  | Diabetes mellitus | 8 (19.5) | 2 (9.5) |
|  | Asthma | 3 (7.3) | 1 (4.8) |
|  | Coronary heart disease | 7 (17.1) | 4 (19.1) |
|  | Chronic kidney disease | 1 (2.4) | 4 (19.1) |
|  | Chronic liver disease | 0 (0.0) | 0 (0.0) |
|  | Active cancer | 4 (9.8) | 3 (14.3) |
|  | Immunodeficiency | 1 (2.4) | 0 (0.0) |
| ICU deaths | | 27 (65.9) | 8 (38.1) |
| Mortality rate (95% CI) per 1000 patient-days | | 41 (28-59) | 23 (12-46) |
| Length of ICU stay, days | | 15 (8-23) | 6 (4-16) |
| Central venous catheter, days | | 16.5 (7-23) | 7 (3.5-21.5) |
| Urinary catheter, days | | 15 (8-23) | 12 (4-18) |
| Invasive ventilation, days | | 9 (6-19) | 5.5 (3-15) |
| Patients with invasive ventilation | | 37 (90.2) | 16 (76.2) |
| Patients with at least one HAI | | 20 (48.8) | 7 (33.3) |
| Patients with at least one dr-HAI | | 16 (39.0) | 4 (19.1) |
| HAI per patient | | 0.78 (0.94) | 0.62 (0.92) |
| dr-HAI per patient | | 0.54 (0.74) | 0.24 (0.54) |
| HAI per infected patient | | 1.60 (0.68) | 1.85 (0.38) |
| dr-HAI per infected patient | | 1.38 (0.50) | 1.25 (0.50) |
| Antibiotic consumption before the first HAI | |  |  |
|  | Carbapenems | 38 (92.7) | 13 (61.9) |
|  | Extended spectrum cephalosporins | 4 (9.8) | 2 (9.5) |
|  | Glycopeptides | 27 (65.9) | 2 (9.5) |
|  | Macrolides | 14 (34.2) | 1 (4.8) |
|  | Penicillins + beta lactamase inhibitors | 26 (63.4) | 5 (23.8) |
| ICU: Intensive Care Unit. HAI: Healthcare-Associated Infection. dr-HAI: device-related Healthcare-Associated Infection. CI: Confidence Interval. | | | |

Table S2. Type and frequency of all healthcare-associated infections (HAIs) registered by the active surveillance system among the patients admitted to the main intensive care unit of Umberto I teaching hospital of Rome between 1^st^ March and 4^th^ April 2020. Results are expressed as number (percentage).

|  | | | 2020 cohort | |
| --- | --- | --- | --- | --- |
|  |  |  | With COVID-19 | Without COVID-19 |
| HAI | | | 32 (100) | 13 (100) |
|  | Device-related HAI | |  |  |
|  |  | VAP | 15 (46.9) | 2 (15.4) |
|  |  | CRBSI | 0 (0.0) | 0 (0.0) |
|  |  | CAUTI | 7 (21.9) | 3 (23.1) |
|  | BUO | | 9 (28.1) | 5 (38.5) |
|  | *Clostridium difficile* infection | | 0 (0.0) | 2 (15.4) |
|  | Surgical site infection | | 0 (0.0) | 1 (7.7) |
|  | Healthcare-associated pneumonia | | 1 (3.1) | 0 (0.0) |
| Microorganism | | | 44 (100) | 18 (100) |
|  | *Acinetobacter baumannii* | | 13 (29.5) | 5 (27.7) |
|  | *Candida albicans or parapsilosis* | | 5 (11.4) | 1 (5.6) |
|  | *Clostridium difficile* | | 0 (0.0) | 2 (11.1) |
|  | *Enterobateriaceae* | | 8 (18.2) | 1 (5.6) |
|  | *Enterococci* | | 2 (4.5) | 1 (5.6) |
|  | *Klebsiella pneumoniae* | | 6 (13.6) | 3 (16.7) |
|  | *Pseudomonas aeruginosa* | | 4 (9.1) | 1 (5.6) |
|  | *Staphylococcus aureus* | | 1 (2.3) | 1 (5.6) |
|  | Coagulase Negative *Staphylococci* | | 5 (11.4) | 3 (16.7) |
| VAP: Ventilator-Associated Pneumonia. CRBSI: Catheter-Related Blood Stream Infection. CAUTI: Catheter-Associated Urinary Tract Infection. BUO: Bloodstream infections of Unknown Origin. | | | | |
